# Placental *PHLDA2* expression is increased in cases of fetal growth restriction following reduced fetal movements

**DOI:** 10.1186/s12881-016-0279-1

**Published:** 2016-03-05

**Authors:** Anna Bugge Janssen, Simon J. Tunster, Alexander E. P. Heazell, Rosalind M. John

**Affiliations:** Cardiff School of Biosciences, Cardiff University, Cardiff, Wales CF10 3AX UK; Maternal and Fetal Health Research Centre, University of Manchester, Manchester, UK

**Keywords:** Reduced fetal movements, Stillbirth, Fetal growth restriction, Placenta, *PHLDA2*

## Abstract

**Background:**

Maternal perception of reduced fetal movements (RFM) is associated with increased risk of fetal growth restriction (FGR) and stillbirth, mediated by placental insufficiency. The maternally expressed imprinted gene *PHLDA2* controls fetal growth, placental development and placental lactogen production in a mouse model. A number of studies have also demonstrated abnormally elevated placental *PHLDA2* expression in human growth restricted pregnancies. This study examined whether *PHLDA2* was aberrantly expressed in placentas of RFM pregnancies resulting in delivery of an FGR infant and explored a possible relationship between *PHLDA2* expression and placental lactogen release from the human placenta.

**Methods:**

Villous trophoblast samples were obtained from a cohort of women reporting RFM (*N* = 109) and *PHLDA2* gene expression analysed. hPL levels were assayed in the maternal serum (*N* = 74).

**Results:**

Placental *PHLDA2* expression was significantly 2.3 fold higher in RFM pregnancies resulting in delivery of an infant with FGR (*p* < 0.01), with highest levels of *PHLDA2* expression in the most severe cases. Placental *PHLDA2* expression was associated with maternal serum hPL levels (*r* = −0.30, *p* = 0.008, *n* = 74) although this failed to reach statistical significance in multiple linear regression analysis controlling for birth weight (*p* = 0.07).

**Conclusions:**

These results further highlight a role for placental *PHLDA2* in poor perinatal outcomes, specifically FGR associated with RFM. Furthermore, this study suggests a potential relationship between placental *PHLDA2* expression and hPL production by the placenta, an association that requires further investigation in a larger cohort.

**Electronic supplementary material:**

The online version of this article (doi:10.1186/s12881-016-0279-1) contains supplementary material, which is available to authorized users.

## Background

Maternal perception of reduced fetal movements (RFM) is associated with an increased risk of poor perinatal outcomes such as fetal growth restriction (FGR), preterm birth, fetal distress, and stillbirth [1–7]. Placental insufficiency, the reduced ability of the placenta to supply nutrients and oxygen to the fetus, could explain the co-occurrence of RFM and FGR [8]. Placental insufficiency is thought to primarily result in asymmetric FGR with prolonged exposure resulting in RFM (as a mechanism for conserving energy for vital functions) and, if this remains undetected, stillbirth [8]. Indeed, altered placental structure and function has been reported in RFM pregnancies [9–12]. For example, RFM placentas are smaller, lighter and exhibit a specific reduction in the syncytiotrophoblast layer of the placenta [9], which synthesises hormones such as human placental lactogen (hPL) for release into the maternal circulation [13].

Imprinted genes are monoallelically expressed with expression depending on the parent of origin [14]. These genes have well-established roles in controlling fetal growth and placental development, and are regulated by epigenetic marks that may respond to environmental stimuli [15–17]. A subset of imprinted genes also converge on the endocrine lineages of the mouse placenta to regulate placental hormone production [18]. The maternally expressed imprinted gene *PLECKSTRIN HOMOLOGY-LIKE DOMAIN FAMILY A MEMBER 2* (*PHLDA2*) exemplifies all of these functions. Importantly, abnormally elevated placental *PHLDA2* expression has been associated with FGR and/or low birth weight in a number of studies (reviewed in [16]). Modelling similar overexpression of *Phlda2* in a mouse model results in FGR [19, 20], inferring that the association observed in human pregnancies is causal. Moreover, the growth restriction which occurs in response to *Phlda2* overexpression is late onset and asymmetric, followed by rapid postnatal catch up growth [20], which is typical of human pregnancies complicated by placental insufficiency. The *Phlda2* overexpression mouse model also exhibits stunted placental growth with a specific reduction in a key endocrine lineage and impaired placental lactogen production [19, 21], consistent with the altered endocrine function observed in human RFM placentas [9, 12].

In this study we analysed *PHLDA2* expression in a cohort of women reporting RFM (*N* = 109). We hypothesised that placental *PHLDA2* expression is specifically increased in RFM pregnancies complicated by FGR (*N* = 20). We also analysed maternal serum hPL levels (*N* = 74) in the RFM cohort to explore the potential link between *PHLDA2* expression and placental lactogen in the human placenta.

## Methods

### Participant recruitment

Study participants included women delivering within one week of presentation with maternal perception of RFM (after 28 weeks gestation) [2, 9]. Written informed consent was obtained from the participants and the study was approved by Oldham and Greater Manchester North Research Ethics Committees (REC no. 08/1011/83 and 11/NW/0664). Fetal growth restriction was defined as customised birth weight centile <10^th^ [2]. Participant demographics are shown in Table 1.

**Table 1:** Participant demographics of RFM participants (*N* = 109)

	Mean (SD)/Range or Number (%)		Mean (SD)/Range or Number (%)
Birth outcomes			
*Fetal sex*		*Ethnicity*	
Male	56 (51 %)	Caucasian	73 (67 %)
Female	53 (49 %)	African/Afro-Caribbean	10 (9 %)
Gestational age *(days)*	278 (13)/211 – 295	Indian/Pakistani/Bangladeshi	21 (19 %)
Birth weight *(g)*	3306 (599)/850 - 4680	Other	5 (5 %)
Custom birth weight centile	40 (29)/0 - 100	Parity	1 (1.22)/0 – 7
Placental Weight *(g)*	587 (127)/353 – 854	*Smoking during Pregnancy*	
Apgar Scores (1 min)	9 (1.66)/0 - 10	Yes	17 (16 %)
Apgar Scores (5 min)	10 (1.39)/0 – 10	No	92 (84 %)
*Mode of Delivery*		*Alcohol consumption*	
SVD	61 (56 %)	None	107 (98 %)
ELCS	10 (9 %)	1-5 units/week	2 (2 %)
EMCS	14 (13 %)	*Obstetric complications*	
Instrumental	24 (22 %)	None	84 (77 %)
Maternal characteristics		Preeclampsia/PIH/Proteinuria	5 (5 %)
Age (years)	29 (5.81)/17 – 46	Repeat RFM	10 (9 %)
Maternal BMI	25.78 (5.26)/17 – 46	Oligohydramnios	1 (1 %)
		Suspected growth restriction	3 (3 %)
		Other	4 (4 %)

Mean (SD)/Range or Number (%)

*RFM* reduced fetal movements, *BMI* body mass index, *PIH* pregnancy induced hypertension

### Sample collection

Villous trophoblast samples were obtained within 30 min of delivery [9]. As intraplacental variation in *PHLDA2* expression has previously been demonstrated [22], samples were consistently taken midway between the cord insertion and distal edge. Maternal venous serum samples were obtained on admission (*N* = 74) and hPL levels measured by ELISA (Immunodiagnostic systems, Boldon, UK) as previously described [2].

### Gene expression analysis

Total RNA was extracted from the placental tissue samples using GenElute Mammalian Total RNA Miniprep Kit (Sigma-Aldrich, Dorset, UK). 5 μg of RNA was reverse transcribed using M-MuLV reverse transcriptase (Promega, Southampton, UK) with random hexamers, according to manufacturer’s instructions. Quantitative RT-PCR was performed using Chromo 4 Four Colour Real Time Detector (MJ Research) in a 20 μl reaction containing 5 μl of cDNA (diluted 1 in 50), 1X Buffer 2 mM MgCl_2_, 2 mM dNTPs, 0.65 Units Taq (Fermentas (Thermo), Loughborough, UK), 1 μM of each primer (Sigma-Aldrich, Dorset, UK) and 0.12X Sybr Green (Invitrogen, Paisley, UK). All samples were run in triplicate and duplicate plates were performed. Conditions for amplification were: 1) 15 min at 94 °C, 2) 30 s at 94 °C, 3) 30 s at 60 °C, 4) 30 s at 72 °C and 5) 30 s 75 °C, with steps 2–5 repeated for a total of 40 cycles. Melt Curve was performed from 70 to 94 °C, reading every 0.5 °C and holding for 2 s. Primer sequences were as follows: *YWHAZ* forward: TTCTTGATCCCCAATGCTTC & reverse: AGTTAAGGGCCAGACCCAGT, *PHLDA2* forward: GAGCGCACGGGCAAGTA & reverse: CAGCGGAAGTCGATCTCCTT [23] and *L19* forward: CCAACTCCCGTCAGCAGATC & reverse: CAAGGTGTTTTTCCGGCATC [24].

### Statistical analysis

Gene expression data is presented as the ∆CT (target gene expression relative to the housekeeping gene *YWHAZ*) and as the fold change in expression, calculated using the 2 ^-∆∆CT^ method [25]. The geometric mean of two housekeeping genes, *YWHAZ* and *L19,* was used to confirm results obtained using the single housekeeping gene, *YWHAZ*, for a subset of samples. Parametric statistical tests were used to analyse normally distributed data. The effect of potential confounders (infant birth weight, gender and gestational age) was examined using multiple linear regression analysis. To ease interpretation, ∆CT values have been inverted [x(−1)] such that lower values represent decreasing gene expression.

## Results

**Fig. 1:**
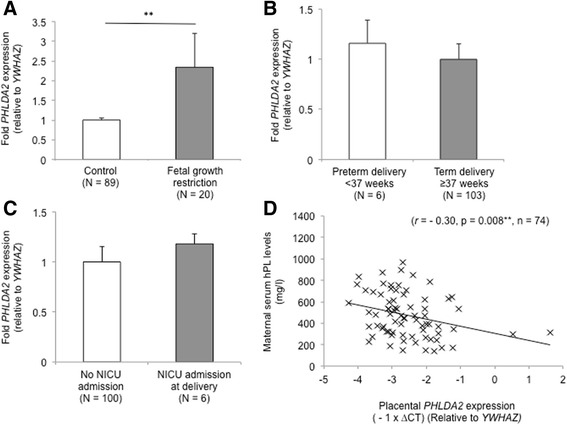
Placental *PHLDA2* expression in RFM pregnancies. Placental *PHLDA2* expression was significantly increased in RFM pregnancies resulting in delivery of a growth restricted infant (**a**) Fetal growth restriction was defined as delivery of a term infant with a custom birth weight centile <10^th^. Placental *PHLDA2* was not significantly altered in RFM pregnancies where infants were born preterm (**b**) or admitted to NICU at delivery (**c**) There was a significant inverse association between placental *PHLDA2* expression and maternal serum hPL levels (**d**) RFM = reduced fetal movements. Error bars represent SEM. ** *P* < 0.01

Placental *PHLDA2* expression was significantly 2.3 fold higher in RFM pregnancies resulting in delivery of a growth restricted compared with a normal birth weight infant (Fig. 1a). Results remained statistically significant when comparing normalisation to a single housekeeping gene *YWHAZ* with the geometric mean of *YWHAZ* and *L19* expression in a subset of samples (Additional file 1: Figure S1).

When participants were further classified according to severity of growth restriction, *PHLDA2* expression was significantly 1.7 fold higher in cases with custom birth weight centile <10^th^ (*p* = 0.01, *n* = 12. v. 82) and 3.2 fold higher in cases with custom birth weight centile <3^rd^ (*p* < 0.001, *n* = 8 v. 82). Placental *PHLDA2* expression was also significantly inversely associated with birth weight (*r* = −0.24, *p* = 0.01, *n* = 109), custom birth weight centiles (*r* = −0.25, *p* = 0.01, *n* = 109) and, in this study, also placental weight (*r* = −0.39, *p* = 0.03, *n* = 31).

We subsequently analysed placental *PHLDA2* expression in relation to preterm delivery and NICU admission at birth, which are other common poor perinatal outcomes associated with RFM. There was no significant correlation between placental *PHLDA2* expression and gestational age (*r* = −1.41, *p* = 0.14, *n* = 109) and was no significant difference in expression between preterm and term deliveries (Fig. 1b). Placental *PHLDA2* expression was not significantly altered in infants admitted to NICU at delivery (for perinatal asphyxia) (Fig. 1c) and there was no significant correlation between placental *PHLDA2* and other measures of infant wellbeing at delivery including Apgar scores at 1 min (*r* = 0.01, *p* = 0.93, *n* = 106) and 5 min (*r* = −0.11, *p* = 0.27, *n* = 105) or with arterial cord blood pH (*r* = − 0.7, *p* = 0.52, *n* = 76).

Imprinted genes have been demonstrated to regulate the endocrine lineage of the mouse placenta, in particular expression of placental lactogens [18]. In this study there was a significant inverse association between placental *PHLDA2* expression and maternal serum hPL levels (Fig. 1d) suggesting that *PHLDA2* may regulate the production of placental hormones in human pregnancies. In further multiple linear regression analysis controlling for infant birth weight, offspring gender and gestational age (F(4,69) = 5.34, *p* = 0.001, *R*^2^ = 0.24), the association between placental *PHLDA2* expression and serum hPL levels failed to reach statistical significance (*p* = 0.07), with only infant birth weight significantly associated with maternal serum hPL levels (*p* = 0.01). This is perhaps not surprising since, in the mouse model, elevated *Phlda2* drives both growth restriction and reduced expression of placental lactogens [19, 21]. Data from the animal model supports a causal association between placental *PHLDA2* expression and maternal serum hPL levels.

## Discussion

A number of studies have demonstrated abnormally elevated placental *PHLDA2* in pregnancies complicated by FGR (reviewed in [16]). The *Phlda2* overexpression mouse model exhibits FGR and impaired placental development supporting a causal role for elevated *PHLDA2* in human FGR [19, 20]. Uniquely, this study examined a group of women reporting RFM, a group who are at high risk of poor perinatal outcomes. Within this group, there was a significant association between increased placental *PHLDA2* expression and FGR. Moreover, the highest levels of *PHLDA2* expression were present in the most severely growth restricted cases which also had the most severe placental phenotype [12]. Placental weight was also significantly inversely associated with *PHLDA2* expression, which has not previously been reported. This is of clinical interest as RFM is thought to represent a fetal adaptation to prolonged placental insufficiency which, if undetected, may result in stillbirth [8]. While all participants in the current study delivered live born infants, increased expression of *PHLDA2* has previously been reported in cases of spontaneous miscarriage or fetal death [26] and more specifically, in cases of fetal death attributed to FGR [27]. These results further highlight a role for placental *PHLDA2* in poor perinatal outcomes.

Our second key finding in this study was the significant association between elevated placental *PHLDA2* expression and decreased maternal hPL serum levels. After controlling for birth weight, this association failed to reach statistical significance (*p* = 0.07) but this can be explained if, as in the mouse [19, 21], elevated *PHLDA2* drives both growth restriction and reduced placental lactogen expression. hPL plays an important role in maternal glucose management during pregnancy and in the control of fetal growth [28]. This relationship between placental *PHLDA2* expression and placental hPL production would therefore be of considerable interest in understanding the mechanisms driving poor growth *in utero*.

## Conclusion

This study demonstrated significantly elevated *PHLDA2* expression in placentas of RFM pregnancies resulting in delivery of a growth restricted infant and identified an association between placental *PHLDA2* expression and serum hPL levels. It has previously been suggested that *PHLDA2* expression in the placenta could be used to postnatally identify types of fetal growth restriction and thus inform subsequent infant care [16]. Identifying hormonal biomarkers of abnormal placental *PHLDA2* expression during pregnancy could be further used as a prenatal diagnostic tool to identify RFM pregnancies at risk of poor perinatal outcomes. This is of particular importance in the context of a recent UK report highlighting that more appropriate, better informed management of RFM could have prevented one third of the stillbirth cases examined [29].

## Availability of data and materials

Cohort data and details on availability of material [2, 9].

## Additional file

Additional file 1: Figure 1. Placental *PHLDA2* expression in RFM pregnancies, normalised to the geometric mean of *YWHAZ* and *L19* expression*. PHLDA2* expression was significantly increased in placenta from pregnancies complicated by fetal growth restriction when normalized to *YWHAZ* expression (A) or to the geometric mean of *YWHAZ* and *L19* expression (B) in a subset of samples from the full cohort (*N* = 37). Expression of the housekeeping genes *YWHAZ* and *L19* was significantly correlated (*r* = 0.94, *p* < 0.001, *n* = 37). There was no significant difference in expression between RFM placenta from normal birth weight pregnancies and pregnancies resulting in fetal growth restriction (*p* = 0.37 and *p* = 0.45 respectively, *n* = 3 (TIF 2931 kb)

## Abbreviations

FGR: feta growth restriction
hPL: human placental lactogen
*PHLDA2*: Pleckstrin homology-like domain family A member 2
RFM: reduced fetal movements
