# Supplementary figures and images for: Placental PHLDA2 expression is increased in cases of fetal growth restriction following reduced fetal movements
Source: BMC Med Genet. 2016 Mar 5;17:17. doi: 10.1186/s12881-016-0279-1 (PMC4779203; doi:10.1186/s12881-016-0279-1)

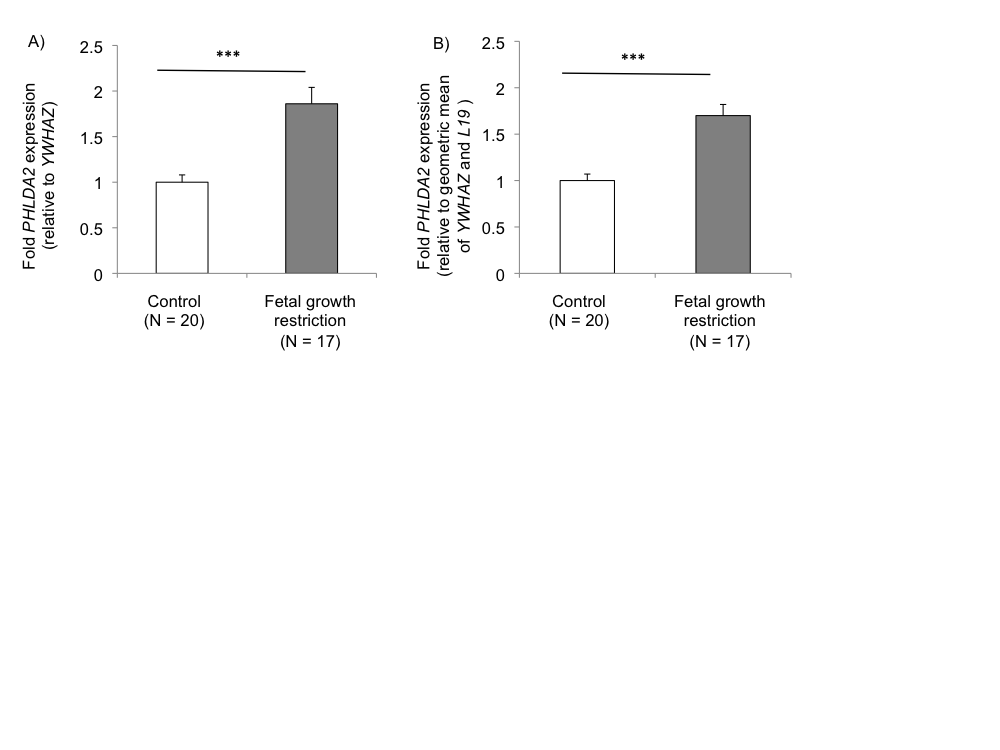

Supplement: Additional file 1: Figure 1. — Placental PHLDA2 expression in RFM pregnancies, normalised to the geometric mean of YWHAZ and L19 expression. PHLDA2 expression was significantly increased in placenta from pregnancies complicated by fetal growth restriction when normalized to YWHAZ expression (A) or to the geometric mean of YWHAZ and L19 expression (B) in a subset of samples from the full cohort (N = 37). Expression of the housekeeping genes YWHAZ and L19 was significantly correlated (r = 0.94, p < 0.001, n = 37). There was no significant difference in expression between RFM placenta from normal birth weight pregnancies and pregnancies resulting in fetal growth restriction (p = 0.37 and p = 0.45 respectively, n = 3 (TIF 2931 kb) [file 12881_2016_279_MOESM1_ESM.tif]
